# Supplementary material for: The MATES Case Management Model: Presenting Problems and Referral Pathways for a Novel Peer-Led Approach to Addressing Suicide in the Construction Industry
Source: Int J Environ Res Public Health. 2021 Jun 23;18(13):6740. doi: 10.3390/ijerph18136740 (PMC8269434; doi:10.3390/ijerph18136740)
Supplement: Supplementary file 1 [file ijerph-18-06740-s001.zip › ijerph-1189834-supplementary.pdf]

Supplementary table 1. Six-item exit survey administered to MATES clients.

Question	Response				
	Absolutely Yes	Yes	Maybe	No	Absolutely No
1) Do you feel the nature of your concerns were met during the case management process?					
2) Were the services appropriate in meeting your needs					
3) Did you feel actively involved in the decision-making process?					
4) Do you consider that your medical, emotional, mental well-being and spiritual needs were addressed?					
5) Would you recommend MATES to co-workers, family and friends?					
6) Any comments about MATES or case management you would like to make:	Open answer response				
